# A Challenging Case of Severe Ulcerative Colitis following the Initiation of Secukinumab for Ankylosing Spondylitis

**DOI:** 10.1155/2018/9679287

**Published:** 2018-02-15

**Authors:** Dean Ehrlich, Nimah Jamaluddin, Joseph Pisegna, David Padua

**Affiliations:** ^1^Division of Digestive Diseases, Department of Medicine, David Geffen School of Medicine at UCLA, Los Angeles, CA, USA; ^2^Division of Gastroenterology, Hepatology and Parenteral Nutrition, VA Greater Los Angeles Healthcare System, Los Angeles, CA, USA

## Abstract

Secukinumab is an interleukin-17 inhibitor used for the treatment of ankylosing spondylitis (AS), psoriasis, and psoriatic arthritis. The risk of exacerbating underlying inflammatory bowel disease (IBD) in patients being treated with secukinumab for other conditions is controversial. We document a patient with AS and previously undiagnosed IBD, found to be in a severe ulcerative colitis flare shortly after receiving the loading dose of secukinumab. There are no guidelines regarding biologic salvage therapy for IBD in the setting of active treatment with another biologic agent. After waiting one half-life of secukinumab, our patient had an excellent response to initiation of infliximab.

## 1. Introduction

Secukinumab is an interleukin-17 (IL-17) monoclonal antibody inhibitor approved for the treatment of ankylosing spondylitis, psoriatic arthritis, and psoriasis [1]. Data previously showed that secukinumab has no efficacy in Crohn's Disease [2]; however, the risk of exacerbating underlying Crohn's Disease or Ulcerative Colitis in patients being treated with secukinumab for other conditions is controversial.

## 2. Case

We present the case of a 42-year-old male with a past medical history of ankylosing spondylitis (AS) and anterior uveitis who came to the hospital with nonbloody diarrhea. For the ten days prior to presentation, the patient reported over twenty loose nonbloody bowel movements per day, with associated nocturnal symptoms, tenesmus, fevers, chills, night sweats, and five-pound weight loss. For the two years prior to the onset of these symptoms, he had five bowel movements per day, with intermittent bloody stool, tenesmus, and 20-pound weight loss but never had a workup for inflammatory bowel disease (IBD) including a prior colonoscopy. He was a resident of Los Angeles with no recent travel outside the continental United States and no unusual exposures. He denied relevant family history of inflammatory disorders. He had attributed these chronic gastrointestinal symptoms to a prior diagnosis of external hemorrhoids.

His ankylosing spondylitis (HLA-B27 positive) was active with severe daily stiffness and thoracolumbar back pain but had no symptoms of anterior uveitis for several years. For AS, he was prescribed naproxen and methotrexate. He had previously been trialed on etanercept and then adalimumab, which were both discontinued due to rash. Six weeks prior to admission, he was started on secukinumab and received all five loading doses without change in his AS symptoms. He stopped taking naproxen 10 days prior to admission when the diarrhea began.

**Figure 1:**
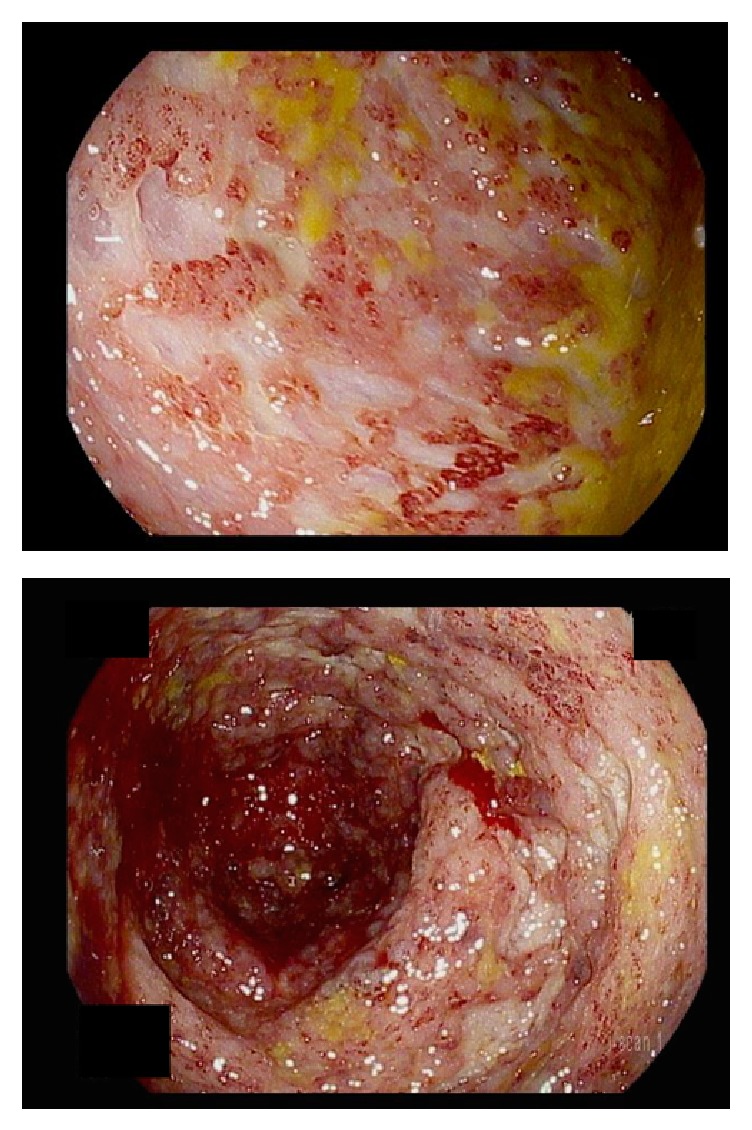
Endoscopic evaluation on presentation. Severe colitis seen throughout the colon.

On admission, his exam was notable for a temperature of 102.3°F and the absence of any eye, oral, skin, or rectal abnormalities. His abdomen was soft but diffusely tender. Labs were notable for 13.99 (M/uL) white blood cells, hemoglobin 10.6 (g/dL), platelets 501 (k/uL), ESR 78 (mm/hr), CRP 18.9 (mg/dL), albumin 3.2 (g/dL), and fecal calprotectin 359 (mcg/g). He had a normal complete metabolic panel and TSH, with negative HIV, blood cultures, and stool infectious workup, including C. diff toxin. A CT abdomen and pelvis with contrast showed pancolitis. A flexible sigmoidoscopy performed shortly after admission showed severe colitis with deep ulcerations, absent vascular pattern, and friable mucosa in the transverse and sigmoid colon (Figure 1). There was mild colitis in the rectum with patchy erythema. Pathology from the transverse and distal colon showed focal neutrophilic cryptitis, crypt microabscesses, and missing crypts, consistent with inflammatory bowel disease. Histology was negative for CMV colitis. Magnetic resonance enterography showed no evidence of small bowel disease.

**Figure 2:**
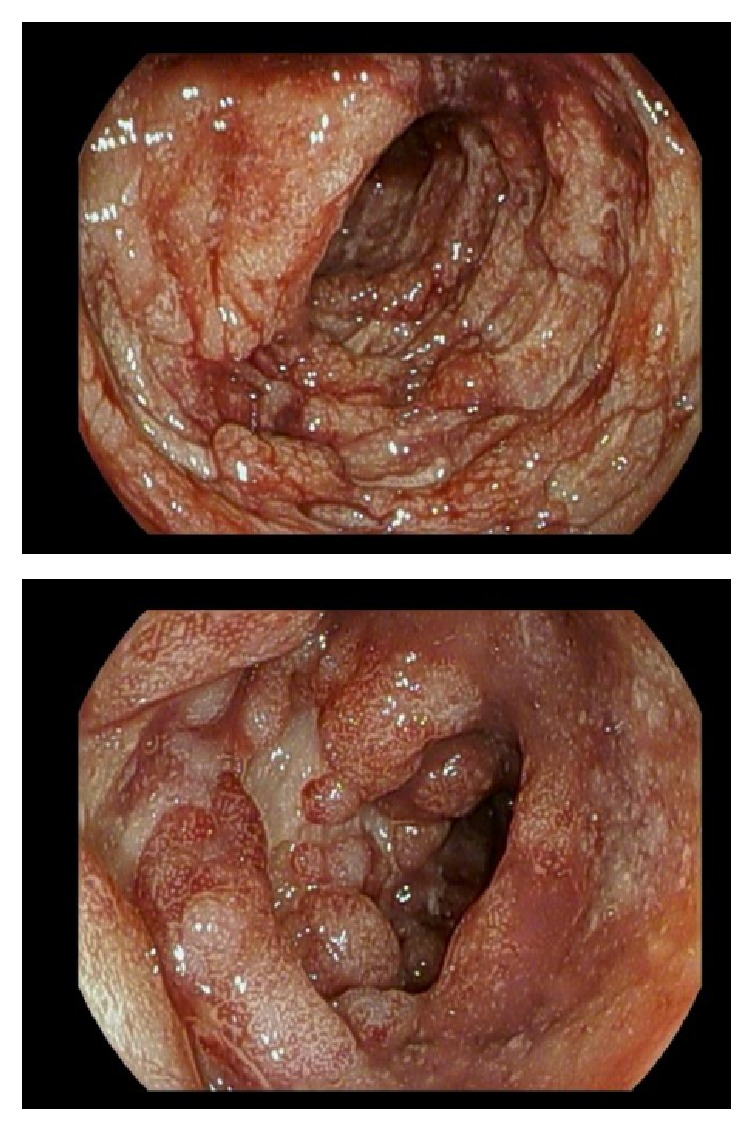
Endoscopic evaluation after failed trial on oral steroids. Severe colitis seen up to 40 cm; further evaluation was limited given degree of inflammation.

A new diagnosis of an ulcerative colitis (UC) flare (Mayo Score 12) was made. The patient was treated with intravenous solumedrol (60 mg per day) and had a brief period of improvement. However, he did not tolerate a transition to oral prednisone (60 mg per day) and suffered a syncopal event from blood loss. A repeat flexible sigmoidoscopy demonstrated edematous mucosa with loss of vascularity and scattered ulcers from anus to 15 cm and deep large ulcers with friable mucosa and spontaneous bleeding from 15 cm to 40 cm (Figure 2).

**Figure 3:**
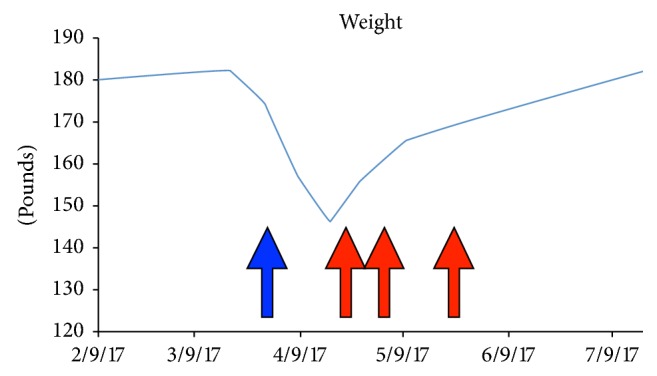
Charted weights. Significant weight loss seen on presentation (blue arrow). Red arrows: indication dates of infliximab administration with the corresponding weight gain.

Surgical consultation was requested after the patient failed the oral steroid trial; however, the patient was hesitant to undergo total proctocolectomy and declined surgical intervention. Biologic salvage therapy was considered, but the timing of initiating a biologic agent was troublesome knowing that he was already on a long acting immunosuppressant (secukinumab) and appreciating the risks of severe immunosuppression. He was put back on intravenous solumedrol; however, his symptoms only mildly improved. After discussion with the patient, the decision was made to start infliximab one month after the last secukinumab infusion along with an oral steroid taper. The patient responded well after two doses of infliximab (10 mg/kg) with improved bloody diarrhea to three times per day and 15-pound weight gain within the first month (Figure 3). The patient has received a total three doses of infliximab. We plan for the patient to receive maintenance infliximab therapy every eight weeks.

## 3. Discussion

This case documents the association of initiating secukinumab, an IL-17 inhibitor, with a severe flare of ulcerative colitis in a patient with previously undiagnosed IBD.

The question of whether secukinumab impacts inflammatory bowel disease first arose in 2012 when a study on the treatment of Crohn's Disease with secukinumab demonstrated poorer outcomes and more adverse events in treated patients than those given a placebo [2]. However, in larger trials on the use of secukinumab in treating AS, there were only six reports of inflammatory bowel disease as adverse events, just two of which were considered serious [3, 4]. Then in October 2016, a review of 14 studies, in which patients were treated with secukinumab for AS, psoriasis, or psoriatic arthritis, made the claim that there was* no* increased risk of IBD due to secukinumab. The review stated that the incidence of IBD in patients treated with secukinumab was similar to rates of IBD seen in the literature in patients with AS, psoriasis, and psoriatic arthritis [5].

The acute management of this patient was challenging because of the recent administration of secukinumab which has a half-life of 22–31 days [1]. The addition of another active biologic agent to treat the IBD flare may have subjected the patient to excessive immunosuppression and placed him at increased risk for a negative outcome. There are no guidelines for management of the waiting period between biologic agents in IBD. In the psoriasis literature, there is some consensus on giving the first dose of a new biologic at the time the next dose of the old biologic would be due; however, this is based on expert opinion [6, 7]. In Crohn's Disease literature, safe administration of adding natalizumab or vedolizumab to infliximab therapy has been described in refractory cases [8, 9]. However, there is no data on using infliximab as an overlapping induction agent in the setting of recent secukinumab use and severe UC. We opted to wait 30 days before starting infliximab (roughly one half-life of secukinumab), which also coincided with the next due dose of secukinumab. Our surgical team was also closely following the patient if his health had declined any further.

An important consideration for this case is that the patient may have had underlying undiagnosed IBD (based on symptom history) and therefore secukinumab may have triggered an IBD flare, rather than a de novo case of IBD. While there is no way to determine whether the patient had previously undiagnosed IBD, even the triggering of an IBD flare by secukinumab has been rarely reported and would be an especially important finding because of the recent literature which specifically refutes this association as a concern for patients with IBD taking secukinumab [5]. Another consideration is that NSAIDs may have triggered the IBD flare; however, the patient had been taking NSAIDs for months to years without issue.

The pathogenesis of UC triggered by secukinumab is largely unknown. Given the delicate balance of the immune response in the setting of inflammatory bowel disease, it is often difficult to predict the effects of cytokine blockade on disease progression. Il-17 antagonists showed promise in preclinical trials. However, not all studies were positive. In 2004, Ogawa et al. reported on a mouse model of colitis that a neutralizing Il-17 monoclonal antibody worsened colitis via increases in CD4-positive helper T cells and CD11b-positive granulocytes-monocytes infiltration and increases in tumor necrosis factor-alpha, interferon-gamma, and IL-6 [10]. It is possible that these mechanisms were at play in our patient. The combination of his potential genetics and secukinumab exposure may have elicited this severe colitis response. Per Hueber et al., in Crohn's Disease genetic polymorphisms (i.e., TL1A) likely play a role in the response to secukinumab [2]. It is unclear whether our patient had this polymorphism; however, this may be important for future risk stratification of patients.

This case presents an interesting study in the potential side effects evoked by IL-17 antagonists. Given the potential risk of IBD exacerbation with these agents, providers should consider evaluating for underlying IBD prior to their initiation. As more targeted therapies come to the market to treat various inflammatory conditions, it will be important to monitor these side effects and develop strategies to ameliorate the adverse events.
